# Quantitative Co-expression and Pathway Analysis Reveal the Shared Biology of Intellectual Disabilities

**DOI:** 10.1192/j.eurpsy.2025.610

**Published:** 2025-08-26

**Authors:** J.-A. Sharif, J. Timmons, P. Chapple

**Affiliations:** 1 Queen Mary University of London, William Harvey Research Institute, London, United Kingdom

## Abstract

**Introduction:**

Intellectual disabilities(ID) are heterogeneous conditions, where mutations in more than 1000 genes are associated with ID. In the Genomic England gene panel, at least 1399 genes have sufficient evidence to be deemed diagnostic grade. The cellular pathways in some of these genes function remain ill-defined.

**Objectives:**

Identification of molecular pathways or upstream regulators common across multiple intellectual disability genes would increase our understanding of the pathophysiological mechanisms and potentially identify therapeutic targets. Quantitative network analysis, using large datasets, is a potentially robust way to identify functional pathways, yet it has not been applied to ID genes at scale.

**Methods:**

We have used co-expression analysis to identify functional modules of genes co-expressed with 1399 causative ID gene in human brain tissues. Specifically, we optimised existing large-scale transcriptomic data from three separate human brain data sets (>1,000 samples), one of which is neocortex (Kang *et al.* Nature 2011; 478 483–489, Trabzuni *et al. Nat Commun*2013; 4, 2771). Optimisation included reannotation, signal filtering, data scaling and then application of Multiscale Embedded Gene Co-expression Network Analysis (MEGENA) framework (Song W-M, Zhang B PLoS Comput Biol 2015; 11 11), to define robust network structures. Gene ontology was carried out using Metascape (Zhou Y *et al.* Nat Commun. 2019 Apr 3;10(1):1523).

**Results:**

Following validation steps, we have confirmed our strategy produced valid co-expression modules for genes with known biology. For example, OXPHOS genes were detected in the same module (Image 1). Moreover, for modules containing ID genes, that have been extensively studied, ontology analysis of the module identified terms related to their known function. A sunburst plot was generated to map intellectual disability genes against co-expression modules (Image 2). The most ID gene enriched neocortex module underwent gene ontology (Image 3) consisting of twelve ID genes, gene ontology was consistent with neuronal cell biology including “trans-synaptic signaling” and “brain development”.

**Image 1:**

**Figure:**
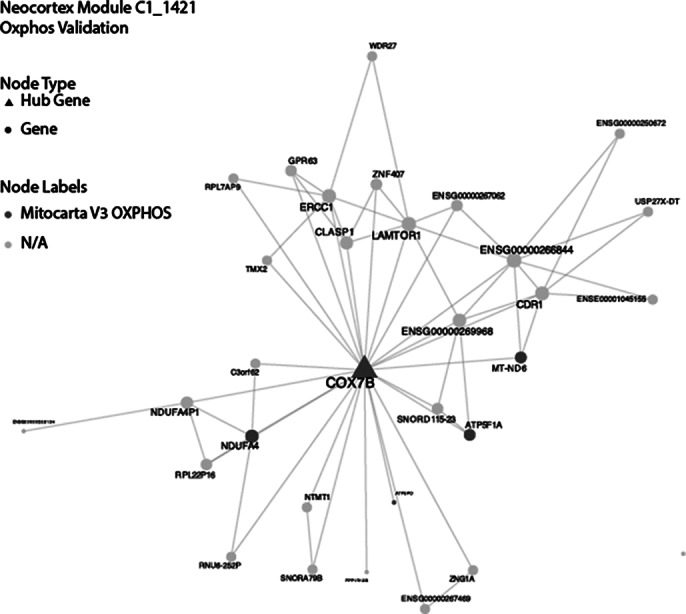

**Image 2:**

**Figure:**
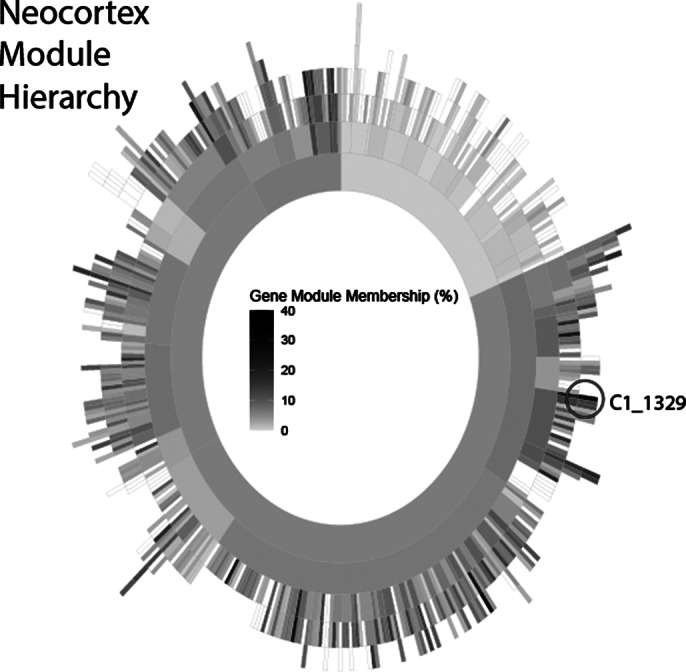

**Image 3:**

**Figure:**
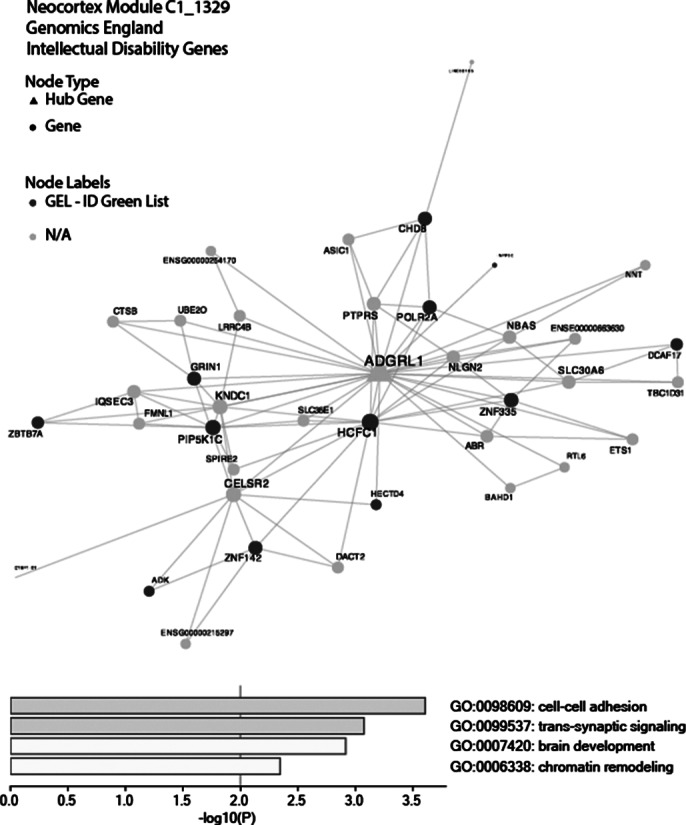

**Conclusions:**

We have identified functional modules of genes that co-expressed with causative ID genes in human brain. The genes HCFC1, ZNF355, GRIN1, POLR2A and PIP5K1C in neocortex module C1_1329 are associated with microcephaly and ID (Koufaris C *et al.*Biomed Rep. 2016, Stouffs K *et al.* Clin Genet. 2018;, Platzer K *et al.* GeneReviews 2019, Haijes HA *et al.* Am J Hum Genet. 2019, Morleo M *et al.* Am J Hum Genet. 2023). Defining ID gene co-expression modules provided new insights into their underlying biology, revealing novel molecular links between different intellectual disability genes. This should improve molecular classification of these diseases as well as highlight therapeutic targets, facilitating drugs discovery and repositioning for rare causes of intellectual disabilities.

**Disclosure of Interest:**

None Declared

